# Quantifying changes in umbilicus size to estimate the relative age of neonatal blacktip reef sharks (*Carcharhinus melanopterus*)

**DOI:** 10.1093/conphys/coad028

**Published:** 2023-05-09

**Authors:** Shamil F Debaere, Ornella C Weideli, Ian A Bouyoucos, Kim B Eustache, José E Trujillo, Gudrun De Boeck, Serge Planes, Jodie L Rummer

**Affiliations:** ECOSPHERE, Department of Biology, University of Antwerp, Groenenborgerlaan 171, 2020 Antwerp, Belgium; Soneva Fushi, Boduthakurufaanu Magu, Male 20077, Maldives; Dr Risch Medical Laboratory, Wuhrstrasse 14, 9490 Vaduz, Liechtenstein; EPHE-UPVD-CNRS, USR 3278 CRIOBE, PSL Research University, Université de Perpignan, 58 Avenue Paul Alduy, 66860 Perpignan Cedex, France; EPHE-UPVD-CNRS, USR 3278 CRIOBE, PSL Research University, Université de Perpignan, 58 Avenue Paul Alduy, 66860 Perpignan Cedex, France; Department of Biological Sciences, University of Manitoba, 50 Sifton Road, Winnipeg, Manitoba R3T 2N2, Canada; ARC Centre of Excellence for Coral Reef Studies, James Cook University, Townsville, QLD 4811, Australia; EPHE-UPVD-CNRS, USR 3278 CRIOBE, PSL Research University, Université de Perpignan, 58 Avenue Paul Alduy, 66860 Perpignan Cedex, France; Institute for Biodiversity and Ecosystem Dynamics, University of Amsterdam, Amsterdam, Netherlands; Department of Marine Science, University of Otago, Dunedin 9016, New Zealand; ECOSPHERE, Department of Biology, University of Antwerp, Groenenborgerlaan 171, 2020 Antwerp, Belgium; EPHE-UPVD-CNRS, USR 3278 CRIOBE, PSL Research University, Université de Perpignan, 58 Avenue Paul Alduy, 66860 Perpignan Cedex, France; Laboratoire d’Excellence ‘CORAIL’, EPHE, PSL Research University, UPVD, USR 3278 CRIOBE, 98729 Papetoai, Moorea, French Polynesia; ARC Centre of Excellence for Coral Reef Studies, James Cook University, Townsville, QLD 4811, Australia; Marine Biology, College of Science and Engineering, James Cook University, Townsville, QLD 4811, Australia

**Keywords:** wound healing, parturition, French Polynesia, elasmobranchs, body condition

## Abstract

Sharks can incur a range of external injuries throughout their lives that originate from various sources, but some of the most notable wounds in viviparous shark neonates are at the umbilicus. Umbilical wounds typically heal within 1 to 2 months post-parturition, depending on the species, and are therefore often used as an indicator of neonatal life stage or as a relative measure of age [*e.g.* grouping by umbilical wound classes (UWCs), according to the size of their umbilicus]. To improve comparisons of early-life characteristics between studies, species and across populations, studies using UWCs should integrate quantitative changes. To overcome this issue, we set out to quantify changes in umbilicus size of neonatal blacktip reef sharks (*Carcharhinus melanopterus*) around the island of Moorea, French Polynesia, based on temporal regression relationships of umbilicus size. Here, we provide a detailed description for the construction of similar quantitative umbilical wound classifications, and we subsequently validate the accuracy of our classification and discuss two examples to illustrate its efficacy, depletion rate of maternally provided energy reserves and estimation of parturition period. A significant decrease in body condition in neonatal sharks as early as twelve days post-parturition suggests a rapid depletion of *in utero*-allocated energy reserves stored in the liver. Back calculations of timing of birth based on the umbilicus size of neonates determine a parturition season from September to January, with most parturitions occurring during October and November. As such, this study contributes valuable data to inform the conservation and management of young-of-the-year blacktip reef sharks, and we therefore encourage the construction and use of similar regression relationships for other viviparous shark species.

## Teaser

Live-bearing shark species are inherently born with an open wound, the umbilicus. The healing status of this umbilicus can be used to estimate the age of newborn sharks. This study proposes the first umbilical wound classification based on quantitative changes in umbilicus size allowing accurate age estimates of newborn sharks.

## Introduction

The world’s oceans are rapidly changing (Halpern *et al.,* 2008; Hoegh-Guldberg and Bruno, 2010; Cooley *et al.,* 2022). Exposure to environmental and anthropogenic stressors, such as those associated with human-driven climate change, fisheries or pollution, may profoundly affect development and growth of elasmobranch fishes (*i.e.* sharks and their relatives) that are critical to the health of marine ecosystems (Rosa *et al.,* 2014; Wheeler *et al.,* 2021). Growth rates make up an important part of a species life history but also represent trade-offs with other life history traits (*e.g.* survival and reproduction). Although a diverse range of growth patterns can be found in sharks and their relatives, they typically exhibit *K*-selected life history strategies, characterized by slow growth rates, late maturation, low fecundity and high longevity (Pianka, 1970; Cortés, 2000). Their slow growth and associated inherent vulnerability to fishing emphasize the importance of understanding these life history characteristics. Indeed, accurate length-at-age descriptions in growth models provide valuable information for assessing population sizes and demographic processes and are crucial to understand productivity, fisheries stock status, maximum sustainable yields and population extinction risks. However, life history data of neonatal sharks, particularly sizes at birth, are often missing from such models, leading to biased growth parameters and rendering their utility less ecologically relevant (Haddon, 2011; Pardo *et al.,* 2013). The collection of morphometric data in neonatal sharks, combined with accurate aging of the neonates, is therefore essential to assess the impacts that chronic stressors may have on future generations, given that early-life stages are some of the most vulnerable.

Fixed length-at-birth (*L*_0_) values based on estimated, as opposed to observed, measurements have frequently been incorporated in growth curves to correct for missing data—often as a result of gear selectivity—on smaller individuals (Pardo *et al.,* 2013). This approach, however, underestimates measures of uncertainty (*e.g.* standard deviations) and can substantially increase growth estimate bias (Pardo *et al.,* 2013). Indeed, sizes at birth are generally highly variable in neonatal sharks, often depending on the amount of energy invested by the mother during embryonic development (Hussey *et al.,* 2010; Weideli *et al.,* 2019a). Moreover, growth rates in the first few years of life are significantly faster than growth rates of adults, with further differences between males and females; yet, adults exhibit some of the slowest growth rates of all vertebrates (Werner and Griebeler, 2014). Such asymmetries discredit a fixed *L*_0_ and preclude accurate length-at-age estimates, thus emphasizing the need to include empirically observed data from neonates to compliment juvenile and (sub)adult growth rates for models that are key to conservation and management (Smart *et al.,* 2015).

Despite substantial knowledge gaps regarding early-life stage morphometric data, it is evident that neonatal sharks have to effectively manage their energy resources to optimize growth while maximizing survival. Structural damage to tissues originating from various sources, including predation and human activities (Chin *et al.,* 2015; Hussey *et al.,* 2017), may however lead to energy being diverted away from routine metabolic activities (*e.g.* growth and foraging) toward healing processes to restore homeostasis. Wound healing is therefore an important process, especially for early-life stages where open wounds are potential sources of infection that may cause complications in neonatal sharks that are still developing their immune system (Chin *et al.,* 2015). Elasmobranchs, in particular, exhibit remarkable wound healing throughout their lives, such as the high healing capabilities observed in juvenile blacktip reef sharks (*Carcharhinus melanopterus*; Chin *et al.,* 2015) and adult whale sharks (*Rhincodon typus*; Womersley *et al.,* 2021), and it is thought that they consistently show a high capacity for wound healing throughout their lives (Chin *et al.,* 2015; Womersley *et al.,* 2021). In viviparous species (*i.e.* those that bear live young), which make up 58% of all elasmobranchs (Compagno, 1990; Dulvy and Reynolds, 1997), the most prominent non-inflicted wounds are at the umbilicus of neonates. Umbilical wounds remain open (*i.e.* underlying muscle tissue remains visible) until ~1–2 months post-parturition, depending on the species’ life history (Castro, 1993; Ulrich *et al.,* 2007; Chin *et al.,* 2015). As such, rapid wound healing at a predictable and consistent site could be used for estimating neonatal life stage and relative age in sharks (Duncan and Holland, 2006; Chin *et al.,* 2015).

The umbilicus has been used extensively to classify neonates of the viviparous Requiem sharks (family Carcharhinidae) into categories based on the umbilical wound healing status (*e.g.*Duncan and Holland, 2006; Aubrey *et al.,* 2007; Hussey *et al.,* 2010; Marie *et al.,* 2017; Weideli *et al.,* 2019a). However, to date, such studies have made use of classifications based on subjective categories (*e.g.* ‘open’, ‘partly healed’ and ‘recently closed’) rather than quantitative changes derived from recaptured individuals. Objective umbilicus size classifications for viviparous shark neonates are needed to interpret and compare early-life characteristics between studies, species, across populations and with respect to environmental and anthropogenic factors. Using more accurate length-at-age data, based on quantitative changes in umbilicus size, instead of pooling length measurements in yearly bins (*e.g.* all neonates and young-of-the-year juveniles in one age-0 group), may in addition considerably increase the model fit of age and growth curves, and hence, their biological and ecological relevance. This may be particularly important for neonatal and juvenile sharks considering their generally high growth rates (Parsons, 1985). Indeed, growth rates decrease monotonically with age when considering a von Bertalanffy growth curve, the most widely applied growth function (Harry *et al.,* 2022).

**Figure 1:**
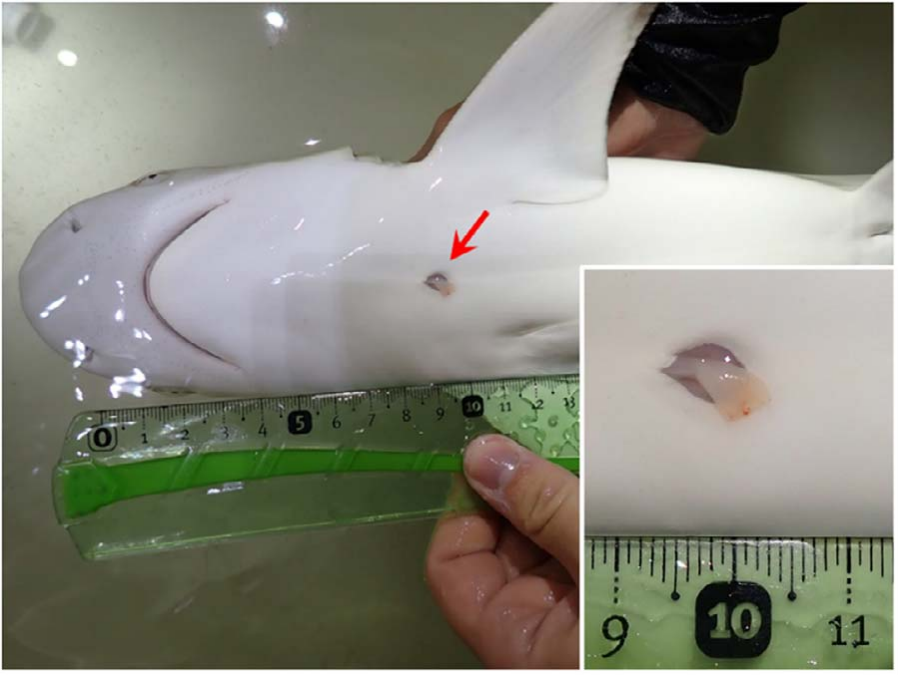
Representative photograph of a neonatal blacktip reef shark with an open umbilical wound (red arrow). In the lower right corner, a close-up of the umbilicus with remnants of the umbilical cord.

The ability to quantitively estimate the age of neonatal sharks may also allow for a more detailed assessment of neonatal mortality rates. Peterson and Wroblewski (1984) developed an age-specific equation to estimate natural mortality rates from weight-at-age data that could be applied to the different umbilical wound classes (UWCs). Indeed, this method has previously been used to estimate the transition in natural mortality rates from young-of-the-year to adult blacktip (*C. limbatus*; Heupel and Simpfendorfer, 2002) and bull (*C. leucas*; Heupel and Simpfendorfer, 2011) sharks; although this indirect technique may underestimate actual mortality rates. Direct estimates through telemetry likely produce more biologically reasonable representations of early-life mortality rates (Heupel and Simpfendorfer, 2002, 2011). A finer resolution of age-specific mortality will further elaborate on the survival dynamics in neonatal sharks. Weight loss during the first few months—post-parturition—has, for instance, been cited as an important cause of neonatal mortality (Duncan and Holland, 2006; Hussey *et al.,* 2010; Corsso *et al.,* 2018; Weideli *et al.,* 2019a). However, the use of subjective categories of umbilical wound healing status and the inconsistency among number of applied classes in previous studies, generally ranging from three to five classes (*e.g.*Duncan and Holland, 2006; Aubrey *et al.,* 2007; Hussey *et al.,* 2010; Marie *et al.,* 2017; Weideli *et al.,* 2019a), make it difficult to compare these data among species and across populations. Furthermore, earlier published qualitative umbilical wound classifications often lack actual ‘time zero’ sharks (*i.e.* neonates bearing remnants of the umbilical cord implying recent birth, hereafter referred to as ‘days-old neonates’; *e.g.*Figure 1), likely due to the small probability of catching these animals within a time span of a few days.

Being able to accurately age neonatal sharks can be a valuable tool for estimating the period and peak of parturition seasons, which represents fundamental knowledge for effective, species-specific fisheries management. However, data on days-old neonates are scarce, and direct observations of parturition in the wild are rare. The period of parturition has therefore often been estimated based on reappearances of newly slender female sharks, for which recent observations indicated pregnancy (Porcher, 2005; Mourier *et al.,* 2013a). However, this method requires intensive monitoring of the adult population via many hours of underwater surveying, and female reappearance may take several weeks. Alternatively, length measurements in combination with embryonic development times and formation of the birth zone in vertebral centra have been used to back calculate the time of parturition (Hall *et al.,* 2012; Santander-Neto *et al.,* 2020). Length measurements are, however, not a suitable indicator of age due to the systematic size overlap across age classes (Weideli *et al.,* 2019a; this study), and studying vertebral centra is only possible in deceased animals. A more convenient and non-lethal method would be to use regression relationships between umbilicus healing rate and the age of neonatal sharks to trace back the timing of parturition.

The primary objective of this study was to quantify changes in umbilicus size of neonatal blacktip reef sharks (*C. melanopterus*) around the island of Moorea, French Polynesia, based on the temporal regression relationships of umbilicus area and perimeter. The abundance of neonatal and juvenile reef sharks on the shallow reefs fringing the island, which serve as parturition areas, make it an ideal location to collect early-life history data and study the population dynamics of these sharks (Mourier and Planes, 2013; Mourier *et al.,* 2013b; Chin *et al.,* 2015; Bouyoucos *et al.,* 2020, 2022). Furthermore, previous research on the wound healing capabilities of blacktip reef sharks make this a well-suited species to address the issues set forth here (Chin *et al.,* 2015), and their extensive distribution throughout the Indo-Pacific (Ebert *et al.,* 2021) may offer the potential for widespread applicability of the proposed classification. The secondary objective was to test and validate the accuracy of our umbilical wound classification to elaborate on the utility of quantitative classifications as the one proposed here, and we provide two examples to illustrate their efficacy. Considering the rapid growth and development of neonatal sharks (Randall, 1977; Gruber and Stout, 1983; Branstetter, 1990; Freitas *et al.,* 2006; Weideli *et al.,* 2019b), neonatal age estimates are crucial data for accurate length-at-age descriptions that are fundamental in understanding productivity, fisheries stock status and risk of population loss and can help further our understanding of early-life growth rates, periods of parturition, initiation of successful feeding, neonatal survival dynamics and the use of parturition areas. As such, this tool provides an absolute needed alternative to lethal sampling for the purpose of age estimates in neonatal sharks.

## Methods and Materials

All shark capture and research protocols were approved under Arrêté N° 9524 issued by the Ministère de la Promotion des Langues, de la Culture, de la Communication et de l’Environnement of the French Polynesian government on 30 October 2015 and James Cook University’s Animal Ethics Committee (A2089, A2394 and A2769). Data were collected over six consecutive parturition seasons (from September to February 2016–2022) as part of long-term, fisheries-independent surveys carried out at the Centre de Recherches Insulaires et Observatoire de l’Environnement (CRIOBE) around Moorea, French Polynesia (17° 30’ S, 149° 50’ W).

**Figure 2:**
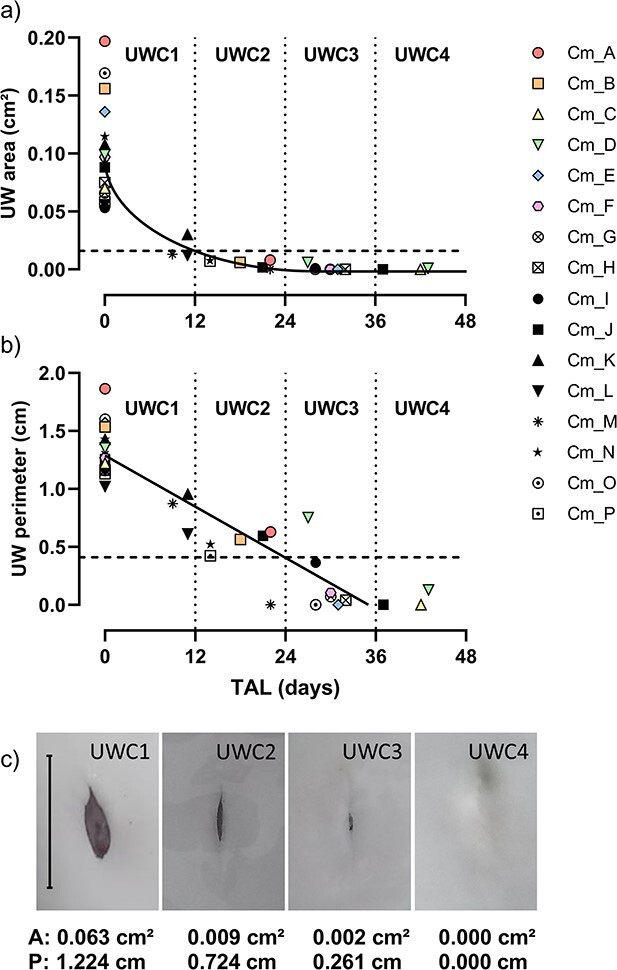
Temporal closing of the umbilicus of neonatal blacktip reef sharks (*n* = 16 unique individuals). (a) Umbilical wound (UW) area of recaptured neonates follows a quadratic decline (*y* = (0.311–0.053 sqrt(*x*))^2^) with a rapid decrease in area during the first 12 days of age. (b) UW perimeter of recaptured neonates follows a negative linear trend (*y* = 1.299–0.037 *x*). The dashed horizontal lines at (a) 0.016 cm^2^ and (b) 0.41 cm represent the critical values at 12 and 24 days of age, respectively, used for the classification of the umbilicus size into four categories, or UWCs (see Table 1). Individuals Cm_D, Cm_J and Cm_M were recaptured twice; the other sharks were only recaptured once. (c) Representative photographs of the umbilicus for each UWC with their corresponding UW area (A) and perimeter (P). Scale bar = 1 cm.

Neonatal and juvenile blacktip reef sharks were caught using a 50 × 1.5 m gillnet with 5-cm mesh size set perpendicular to shore. Gillnets were set at dusk from ~1700 to 2000 h at ten sites (Apaura, Haapiti, Maharepa, Paorea, Papetoai, Pihaena, Tiki, Vaiane, Vaiare and Valorie). Sites were evenly spread out around the 60-km coastline of Moorea, with each site sampled twice per month (*e.g.*Mourier and Planes, 2013; Mourier *et al.,* 2013b; Chin *et al.,* 2015; Bouyoucos *et al.,* 2020, 2022). On capture, sharks were tagged with internal passive integrated transponder (PIT) tags and/or external T-bar anchor tags to allow for the identification of previously caught sharks, their umbilicus was photographed and morphometrics were taken. A ruler was photographed beside each umbilicus for scale (see Figure 1). For the purpose of this study, sharks that have an open umbilicus (*i.e.* with visible muscle tissue, see Figure 2c) are referred to as neonates, whereas those with a fully healed umbilicus (*i.e.* completely closed skin) are referred to as (young-of-the-year) juveniles.

### Quantifying changes in umbilicus size

Sixteen individual days-old neonates (‘time zero’ animals) caught between September 2016 and February 2022—of a total of 727 caught individuals—that were subsequently recaptured during the following weeks were considered for quantifying changes in umbilicus size over time. Neonatal blacktip reef sharks lose the remnants of their umbilical cord within the first couple of days post-parturition (S. Debaere, personal observation), and these remnants are therefore a good indicator of recent birth. To assess the size of the umbilicus of neonatal sharks, photographs were imported in the open-source image processing package Fiji by ImageJ (version 2.0.0-rc-69/1.53c; Schindelin *et al.,* 2012). Scale was set to 1 cm using the ruler in the photographs, and the circumference of the umbilical wound was carefully traced using the polygon selection tool from ImageJ to calculate umbilicus area and perimeter. Temporal closing of the umbilicus of the days-old neonates and their subsequent recapture (13 individuals were recaptured once, 3 individuals were recaptured twice) allowed for a quantitative classification of the umbilicus of the sharks into four categories, or UWCs, based on the area and perimeter of the umbilicus at four time intervals. These four UWCs include three distinct neonatal UWCs (*i.e.* open/unhealed umbilicus, in order of increasing age: UWC1, UWC2 and UWC3) and one juvenile class (*i.e.* closed/healed umbilicus: UWC4).

### Morphometrics and body condition

Morphometric data were collected to assess transitions in growth and body condition across UWCs to shed light on early-life growth patterns and energy reserve depletion rates. Morphometric measurements taken during collections (see preceding section) included total body mass (*M*, in kilograms) and precaudal length (PCL, distance from the tip of the snout to the origin of the caudal fin, in centimeters). In addition, Fulton's body condition factor (*K*, *sensu*Ricker, 1975), derived from the length–mass relationship of the sharks, was calculated as follows:

(1) \begin{equation*} K={10}^5\ M\ {\left(\mathrm{PCL}\right)}^{-3} \end{equation*}

Before statistical analyses, where applicable, data were checked for normality using the Shapiro–Wilk test, where the test statistic W < 0.90 was considered as the critical value to reject the null hypothesis that the data come from a normal distribution. The critical value of statistical significance was set to α = 0.05, and all statistical analyses were carried out in RStudio (version 1.3.1093; RStudio Team, 2020; R Core Team, 2020) using core R packages. We used one-way analyses of variance (ANOVAs) to test for significant differences in body mass, precaudal length or body condition among UWCs. When the initial ANOVA found a statistically significant difference in means, the response variables were compared among the four UWCs using Tukey honest significant difference (HSD) test for multiple comparison.

Note that no discrimination was made between sex because blacktip reef sharks do not sexually mature until they reach a total body length of 105–133.5 cm (between the ages of 4 and 8 years, males and females, respectively) (Stevens, 1984; Chin *et al.,* 2013; Mourier *et al.,* 2013b). Furthermore, previous studies found no significant difference in PCL between male and female neonatal and juvenile blacktip reef sharks (Stevens, 1984; Papastamatiou *et al.,* 2009).

### Back calculating time of parturition

The approximate date of parturition was back calculated for 452 neonatal sharks by subtracting the mean estimated age at the given UWC from the initial capture date using the formula:

(2) \begin{equation*} {t}_0={t}_i-\left({t}_{\max \left({\mathrm{UWC}}_i\right)}-{t}_{\min \left({\mathrm{UWC}}_i\right)}\right)/2 \end{equation*}

where *t*_0_ is the estimated date of parturition (in Julian days), *t_i_* is the initial capture date (in Julian days) and *t*_min(UWC*i*)_ and *t*_max(UWC*i*)_ are the ages (in days) at the lower and higher bounds of the UWC assigned to the shark on initial capture (as provided in Table 1, column ‘Estimated age’). Sharks with a closed umbilicus at initial capture (*i.e.* juveniles) were not considered in these calculations. The distribution of number of parturitions was compared among months using Pearson chi-square test.

**Table 1:** Classification of umbilicus size of neonatal blacktip reef sharks into four UWCs based on temporal changes in UW area and perimeter. Relative age was estimated using the temporal regression relationships obtained for UW area and perimeter presented in Figure 2.

UWC	Estimated age (days)	UW area (cm^2^)	UW perimeter (cm)
1	0–12	>0.016	>0.41
2	12–24	≤0.016	>0.41
3	24–36	<0.016	≤0.41
4	<36	0	0

## Results

### Quantifying changes in umbilicus size

Temporal closing of the umbilicus of recaptured neonates (*n* = 16) suggests a quadratic decline of umbilicus area (fit using the linear mixed-effects *lmer* function from the *lme4* package, with shark identity as random factor, after square root transformation; Bates *et al.,* 2015; Figure 2a) and a negative linear relationship between umbilicus perimeter and time at liberty (TAL) (fit using the *lmer* function with shark identity as random factor; Figure 2b). Based on these regression relationships, umbilical wounds close completely by 36 days post-parturition (see x-intercept in Figure 2b). Critical values for umbilicus area and perimeter were subsequently chosen at 12, 24 and 36 days to obtain a four-point classification with similar time intervals (Figure 2, Table 1). Note from Table 1 that both parameters are needed to assign a UWC to an individual. The rapid decline in umbilicus area allows for distinguishing UWC1 animals from other classes, but umbilicus area appears to overlap between UWC2 and UWC3. The use of umbilicus perimeter is therefore required in addition to area measurements to distinguish the latter two classes.

To validate the accuracy of the temporal regression analyses, the obtained equations were transformed to allow age to be estimated, in days, for a subset of recaptured sharks (via umbilicus area: *age* = [−(sqrt(*area*)−0.311)/0.053]^2^; via umbilicus perimeter: *age* = [*perimeter*−1.299]/[−0.037]). The estimated age of neonates (*i.e.* the mean of the ages inferred from the temporal regression relationships of umbilicus area and perimeter) at initial capture and recapture was subsequently used to predict the elapsed time between initial capture and recapture, referred to as the predicted time at liberty (pTAL). The pTAL was then compared with the actual TAL to validate the accuracy of the temporal regression analyses. Individuals with a closed umbilicus at initial capture and/or recapture and those that were used to construct the regression relationships were omitted from this comparison. On average, the pTAL differed from the actual TAL by 5 ± 3 days (mean ± standard deviation; *n* = 17) (Table 2).

**Table 2:** Validation of the accuracy of the constructed UWC. For a subset of recaptured sharks, the pTAL was calculated from the temporal regression relationships of umbilicus area and perimeter. Actual TAL between initial capture and recapture was subsequently compared to this pTAL value (|TAL − pTAL|). The mean difference (± standard deviation) between TAL and pTAL is provided in the final row.

Shark	Initial capture date	Recapture date	TAL (days)	pTAL (days)	|TAL – pTAL| (days)
1	18/11/2016	02/12/2016	14	18	4
2	20/11/2016	04/12/2016	14	9	5
3	01/12/2016	10/01/2017	9	10	1
4	22/12/2016	07/01/2017	16	8	8
5	06/12/2017	22/12/2017	16	14	2
6	06/12/2017	22/12/2017	16	13	3
7	17/01/2018	26/01/2018	9	13	4
8	13/11/2019	04/12/2019	21	10	11
9	04/12/2019	12/12/2019	8	14	6
10	04/12/2019	12/12/2019	8	12	4
11	06/10/2020	22/10/2020	16	19	3
12	06/10/2020	22/10/2020	16	7	9
13	05/10/2021	26/10/2021	21	12	9
14	11/10/2021	25/10/2021	14	15	1
15	12/11/2021	26/11/2021	14	22	8
16	30/11/2021	28/12/2021	28	25	3
17	06/12/2021	20/12/2021	14	16	2
5 ± 3					

### Morphometrics and body condition

**Figure 3:**
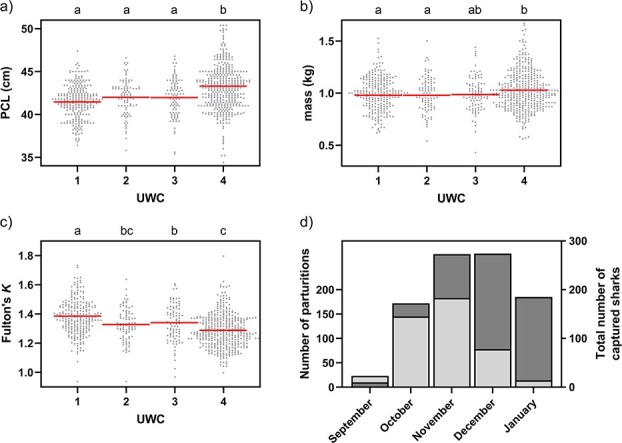
Trends in morphometrics of blacktip reef sharks caught over six consecutive parturition seasons (2016–2022) with increasing UWC. (a) PCL and (b) mass showed a statistically significant positive association with increasing UWC, whereas (c) body condition (Fulton's *K*) significantly decreased with UWC. Letters on top of each panel indicate significant differences among UWCs. Horizontal red lines represent the means of each UWC. Sample sizes (a–c): UWC1 *n* = 254, UWC2 *n* = 93, UWC3 *n* = 96, UWC4 *n* = 407. (d) Distribution of the number of documented blacktip reef shark parturitions throughout the season, as back calculated from the umbilicus size of neonates (left *y*-axis, light grey bars) and the total number of sharks caught each month (right *y*-axis, dark grey bars).

A total of 850 neonatal and juvenile blacktip reef sharks (727 unique individuals) were sampled and measured throughout the six parturition seasons and subsequently assigned a UWC (UWC1 *n* = 254, UWC2 *n* = 93, UWC3 *n* = 96, UWC4 *n* = 407). PCL ranged from 35.4 to 47.4 cm (41.7 ± 2.0 cm) and 34.4 to 57.0 cm (43.3 ± 3.0 cm) in neonates and juveniles, respectively. Mass ranged from 0.430 to 1.525 kg (0.995 ± 0.162 kg) and 0.560 to 2.400 kg (1.055 ± 0.251 kg) in neonates and juveniles, respectively. Both PCL (*F*_3,845_ = 28.78, *P* < 0.001; UWC1–UWC4, *P* < 0.001; UWC2–UWC4, *P* < 0.001; UWC3–UWC4, *P* < 0.001; Figure 3a) and mass (*F*_3,817_ = 5.66, *p* < 0.001; UWC1–UWC4, *P* = 0.003; UWC2–UWC4, *P* = 0.048; Figure 3b) showed a positive relationship with increasing UWC, albeit non-significant among the first three UWCs, whereas body condition (Fulton's *K*; *F*_3,816_ = 25.57, *P* < 0.001; UWC1–UWC2, *P* = 0.003; UWC1–UWC3, *P* = 0.030; UWC1–UWC4, *P* < 0.001; UWC3–UWC4, *P* = 0.009; Figure 3c) significantly decreased with UWC.

### Back calculating time of parturition

The approximate date of parturition was back calculated for neonatal sharks (*i.e.* UWC1–3) by subtracting the mean estimated age at a given UWC from the initial capture date (*i.e.* UWC1 – 6 days; UWC2 – 18 days; UWC3 – 30 days). We observed a clear association between parturition month and the number of neonatal sharks (χ^2^ = 249.13, *P* < 0.001; Figure 3d, left *y*-axis, light grey bars). For the blacktip reef shark population around the island of Moorea, the parturition season starts in September (5.2% of total parturitions), but most parturitions occur during October (32.7%) and November (41.3%), after which the number of births decreases during December (17.6%), and only a few neonates were assumed to have been born in January (3.2%). A similar trend but with 1-month delay can be observed in the total number of sharks caught throughout the parturition season (Figure 3d, right *y*-axis, dark grey bars).

## Discussion

The present study proposes the first umbilical wound classification based on quantitative changes in umbilicus size, a non-lethal alternative to aging neonatal sharks, thus allowing the relative age of neonatal sharks to be estimated. This precise tool will help estimate neonate abundances and inform the use of spatial or temporal fishery closures. Accurate age estimates of neonatal sharks are essential data to further our understanding of their early-life characteristics and environmental and anthropogenic impacts thereupon. As such, this study contributes valuable data to inform the conservation and management of young-of-the-year blacktip reef sharks in French Polynesia, information which could be applied to other populations and species globally. The temporal regression relationships provided in this study for umbilicus area and perimeter are similar to the changes in umbilicus size reported by Chin *et al.* (2015) on studying umbilical wound healing of blacktip reef sharks in a laboratory setting. However, Chin *et al.* did not follow umbilical wound healing in days-old neonates, thus preventing the use of their data for estimating age. Yet, the ability to quantitatively estimate the age of neonatal sharks is fundamental knowledge that can help infer the depletion rate of maternally provided *in utero* energy reserves, subsequent onset of successful feeding and the timing of parturition, as well as many other milestones that are key to early-life history.

Learning how to forage effectively is one of the primary challenges neonatal sharks encounter after parturition (or hatching in oviparous species). Viviparous sharks receive maternal energy reserves in the form of enlarged livers that sustain neonates early in life (Hussey *et al.,* 2010). Once these reserves are depleted, the sharks must start feeding to compensate for the energetic costs of life. Our data show a marked decrease in body condition after age of 12 days (UWC2), suggesting depletion and insufficient replenishment of these energy reserves. Indeed, previous studies have already demonstrated low food consumption rates in coastal young-of-the-year sharks and a reduction in body condition during the first weeks to months post-parturition (Bush and Holland, 2002; Lowe, 2002; Duncan and Holland, 2006; Weideli *et al.,* 2019a). Weideli *et al.* (2019a) reported that the rate of successful feeding (based on the proportion of empty stomachs to stomachs containing prey items) increases with umbilical wound stage (UWC1, 30% of the stomachs contained prey items; UWC2, 47% of the stomachs contained prey items and UWC3, 51% of the stomachs contained prey items), but their data also clearly demonstrate that the sharks seem to feed at insufficient rates (*e.g.* due to limited prey availability), making it difficult in this case to inform on the onset of successful feeding. However, in areas where prey items are abundant and easily accessible, the onset of successful feeding can be more readily quantified.

Similar to our results, Weideli *et al.* (2019a) found a significant decrease in body condition with increasing UWC in the same study population around the island of Moorea but only between young-of-the-year juveniles (*i.e.* sharks with fully healed umbilicus) and first-class neonates (*i.e.* sharks with fully open umbilicus). The quantitative four-point classification applied in our analyses (*i.e.* UWC1–4), as opposed to the qualitative three-point classification used by Weideli *et al.* (2019a) (where UWC1 corresponds to individuals with a ‘fully open umbilicus’; UWC2, ‘semi-healed umbilicus’ and UWC3, ‘fully healed umbilicus’), allowed for more precision in transitions in body condition and suggests a decrease in condition occurs as early as 12 days post-parturition. The *in utero*-allocated energy reserves, therefore, likely only sustain the neonates during the first 2 weeks, rather than month(s), of their lives (cf. depletion of maternal energy reserves in dusky sharks, *C. obscurus*, in Hussey *et al.,* 2010; however, these depletion rates are likely species- and context-specific). A further decrease in body condition observed in the juvenile age class (UWC4) may be the result of limited prey availability or quality, variable foraging strategies and the negative allometric growth reported for the species (*i.e.* faster increase in length than in mass; Weideli *et al.,* 2019a).

We observed the largest variations in PCL, mass and body condition in the juvenile age class (UWC4). The UWC4 comprises all young-of-the-year sharks with a healed umbilical wound, and the time interval in this class is therefore much larger (*i.e.* a resolution of months) than that of UWC1–UWC3 (*i.e.* 12 days each). Animals born during a previous parturition season (*i.e.* +1-year-old sharks) were rarely recaptured (*i.e.* only 6 sharks were recaptured in a subsequent season, based on tag presence) and were therefore excluded from the young-of-year age class. The large variation in morphometric data in UWC4 are therefore likely a result of the variable growth rates in this class (Weideli *et al.,* 2019a, 2019b). In study populations where recapture rates are high, estimates of neonatal age may allow juvenile age of recaptured individuals to be inferred from the elapsed TAL since initial capture, and thus allow for distinct classifications of juvenile groups. However, in our study population around Moorea, we had a recapture rate of ~8.5%, which was too low to get sufficiently large sample sizes if we were to split the juvenile group into more defined classes with similar time intervals to UWC1–3 (*i.e.* ~12 days). The low chance of recapturing individuals on the reefs that fringe Moorea may be due to high neonatal mortality rates together with an expansion in foraging area as the sharks grow. High recapture rates that would allow for distinct juvenile age classes may be more easily obtained from naturally enclosed parturition areas (*e.g.*Gruber *et al.,* 1988, 2001) and something to consider for future investigations and for other species. In addition, in areas where recapture rates are inherently low, neonatal sharks with an actively healing umbilicus could be maintained in sea pens to track healing rates to allow for the construction of similar temporal regression relationships of umbilicus size.

Determining the timing of parturition is critical information for the effective management of shark populations and can be done if neonatal shark ages can be estimated. Back calculating the time of birth in this study suggests that the parturition season of the blacktip reef sharks around the island of Moorea starts in September and lasts until January, with most parturitions occurring during October and November. Our data for Moorea’s blacktip reef shark population provide similar results to those found by Porcher (2005) regarding the progression of adult females through pregnancy, further corroborating the efficacy of our classification.

The accuracy of the proposed umbilical wound classification is further supported by the predictions of elapsed time between initial capture and recapture (pTAL) inferred from the age of the sharks. The error rate of pTAL relative to the actual elapsed TAL of 5 ± 3 days is well within range of our proposed UWCs with a resolution of 12 days. Nevertheless, considering the rapid growth and development of neonatal sharks (Randall, 1977; Gruber and Stout, 1983; Branstetter, 1990; Freitas *et al.,* 2006; Weideli *et al.,* 2019b), an error rate of ~1 week may be considerable. Indeed, a lot may happen during the first weeks of a shark’s life, from learning how to forage (Guttridge *et al.,* 2009; Weideli *et al.,* 2019a), evade predation (Guttridge *et al.,* 2012; Hussey *et al.,* 2017; Trujillo *et al.,* 2022), compete with other neonates (Matich *et al.,* 2017) and cope with anthropogenic stressors (Knip *et al.,* 2010)—stressors that may, in turn, affect healing rates—to the active development of their immune system (Rumfelt, 2014). We therefore encourage the use of UWCs, rather than days-of-age values, to minimize the impact of these error rates.

It is also important to note that umbilical wound classification is likely species- (Castro, 1993; Ulrich *et al.,* 2007) and region-specific because physiological processes of ectotherms, such as wound healing, directly depend on ambient temperature regimes (*e.g.*Anderson and Roberts, 1975; Smith *et al.,* 1988; Pressinotti *et al.,* 2013; Jensen *et al.,* 2015). Higher environmental temperatures, up to the point of thermal stress, may accelerate umbilicus healing rates (Chin *et al.,* 2015, Debaere *et al.*, unpublished data) and thereby influence the regression lines. We therefore encourage comparisons of umbilical wound healing rates across populations to inform the potential for widespread applicability of the proposed classification. To elaborate on the differences in umbilicus healing rates among species, we strongly recommend similar regression relationships as those provided here to be constructed for other viviparous shark species.

In summary, this study is the first to propose an age classification for shark neonates based on quantitative changes in umbilicus size. Our temporal regression relationships of umbilicus area and perimeter allow the relative age of neonatal blacktip reef sharks to be estimated and grouped into distinct UWCs. The accuracy of our umbilical wound classification is supported by the minimal error rates observed between predicted and actual TALs of recaptured neonates. Nevertheless, considering the rapid development of neonatal sharks, we encourage the use of distinct UWCs, rather than their actual age, to minimize the impact of these error rates. Neonatal age estimates are essential data for accurate length-at-age descriptions that are fundamental for understanding productivity, fisheries stock status and population extinction risks. Therefore, these data can help further our understanding of early-life growth rates, neonatal survival, use of parturition areas, essential habitats and ontogenetic shifts in home ranges. Indeed, our data illustrate the efficacy of quantitative classifications of umbilical wound healing status for inferring periods of parturition and highlight how rapidly maternal energy reserves that were provided *in utero* deplete and the delayed onset of successful feeding. Overall, this study contributes valuable data to inform the conservation and management of young-of-the-year blacktip reef sharks in French Polynesia and provides a detailed description for the construction of similar quantitative UWCs for other species.

## Funding

This project was supported by the Laboratoire d’Excellence “CORAIL”, the Station d’Écologie Expérimentale of the CRIOBE, and the French Ministère de l’Environnement. S.F.D. was supported by a University of Antwerp Umbrella Grant and received funding from the Company of Biologists (JEBTF-2105547) and Flying Sharks. O.C.W. received funding from the Save Our Seas Foundation (Keystone Grant no. 290; 2014–2017) and was supported by the Basler Stiftung für biologische Forschung. I.A.B. received funding from the Australian Research Council (ARC) Centre of Excellence for Coral Reef Studies, the Company of Biologists (JEBTF-170510), the British Ecological Society, Passions of Paradise, the Oceania Chondrichthyan Society, and Europcar Polynésie. J.E.T. was supported by a University of Otago Doctoral Scholarship and received funding from the Company of Biologists (JEBTF-1908271) and Flying Sharks. J.L.R. received funding from the Australian Research Council (ARC) Centre of Excellence for Coral Reef Studies, a L’Oréal-UNESCO Women in Science Foundation Fellowship (2015–2016) and an ARC Discovery Early Career Researcher Award (PDE150101266).

## Data Availability Statement

The data underlying this article are available in the Zenodo repository, at https://dx.doi.org/10.5281/zenodo.7232179.

## Author Contributions

S.F.D. conceived the study; S.F.D., O.C.W., I.A.B., K.B.E., J.E.T., G.D.B. and J.L.R. collected field data; S.F.D. analysed the data and drafted the manuscript. All authors secured funding to support this study, contributed to the editing of the final manuscript, and gave final approval for publication.
